# New *Cantharellus* species from South Korea

**DOI:** 10.3897/mycokeys.76.58179

**Published:** 2020-12-22

**Authors:** Bart Buyck, Valérie Hofstetter, Rhim Ryoo, Kang-Hyeon Ka, Vladimír Antonín

**Affiliations:** 1 Institut de Systématique, Écologie, Biodiversité (ISYEB), Muséum national d’histoire naturelle, CNRS, Sorbonne Université, EPHE, 57 rue Cuvier, CP 39, F-75005, Paris, France Muséum national d’histoire naturelle Paris France; 2 Département fédéral de l’économie, de la formation et de la recherche DEFR, Agroscope Domaine de recherche Protection des végétaux, Route de Duillier 60, CP 1012, 1260, Nyon 1, Switzerland Agroscope Domaine de recherche Protection des végétaux Nyon Swaziland; 3 Department of Forest Bioresources, National Institute of Forest Science, Suwon 16631, South Korea National Institute of Forest Science Suwon South Korea; 4 Department of Botany, Moravian Museum, Zelný trh 6, CZ-659 37, Brno, Czech Republic Moravian Museum Brno Czech Republic

**Keywords:** ITS, morphology, new species, phylogeny, *tef*-1

## Abstract

In this third contribution involving new *Cantharellus* species from South Korea, two new species are introduced. In addition, we document a first report of the recently described Japanese *Cantharellus anzutake* outside of Japan based on identical ITS sequence data. *Cantharellus citrinus***sp. nov.** is introduced as a new member of subgenus Cinnabarini, to which the closely related Korean *C. albovenosus* and Chinese *C. phloginus* also belong. *Cantharellus curvatus***sp. nov.** is introduced as a new member of subgenus Parvocantharellus, in which the Korean *C. koreanus* was recently placed. The respective placements of the new taxa are significantly supported by a phylogenetic analysis of sequences from the transcription elongation factor (*tef*-1).

## Introduction

In our previous contributions reporting the biodiversity of *Cantharellus* Adans.:Fr. in Korea (Antonín et al. 2017; Buyck et al. 2018), we have reviewed the still limited taxonomic knowledge on *Cantharellus* species in Asia. During the past two years two more new chanterelles have been described from Asia: *C. anzutake* W. Ogawa, N. Endo, M. Fukuda and A. Yamada from Japan (Ogawa et al. 2018) and *C. hainanensis* N.K. Zeng, Zhi Q. Liang & S. Jiang from China (An et al. 2017). In the present paper, we describe two more new species from South Korea supported by morphological features and in particular by sequence data obtained for the transcription elongation factor (*tef*-1) gene. In addition, identical ITS sequence data also document the presence, in South Korea, of the recently described *C. anzutake* from Japan, a species belonging to subgenus Cantharellus and based on a 100 base pair deletion in the internal transcribed spacer 1 (ITS1) of the rDNA (Ogawa et al. 2018). Obtained *tef*-1 sequence data from all of our recent collections of chanterelles in South Korea could not confirm the presence of any of the European or North American *Cantharellus* previously reported from South Korea (Park and Lee 1991; Kim 2004; Kim et al. 2006; Lee 2011) nor any of the chanterelles already described from India (Das et al. 2015; Kumari et al. 2011, 2013), neighbouring China (Shao et al. 2011, 2014, 2016a, b; Tian et al. 2012; An et al. 2017), Japan (Suhara and Kurogi 2015; Ogawa et al. 2018) or Malaysia (Corner 1966; Eyssartier et al. 2009)

## Materials and methods

### Field work

**Figure 1.**
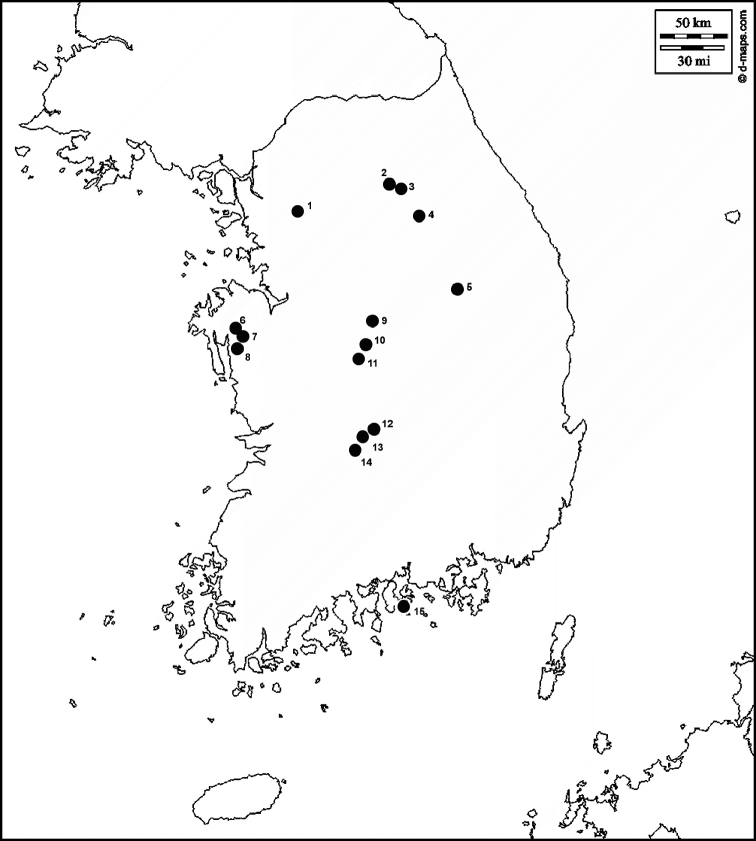
Map of localities of the *Cantharellus* species **1** Hongneung Arboretum, Seoul **2** Suta-sa, Dong-myeon, Hongcheon **3** Experimental Forest, Dong-myeon, Hongcheon **4** Mts. Chiaksan, Haggok-ri, Wonju **5** Guin-sa, Mts. Sobaek, Danyang **6** Yonghyeon National Natural Recreation Forest, Unsan-myeon, Seosan **7** Mts.Gaya, Deoksan-myeon, Yesan **8** Sudeok-sa, Deoksan-myeon, Yesan **9** Mt. Gunjusan, Chilseon-myeon, Goeisan **10** Cheoncheon-myeon, Geoisan **11** Songnisan National Park, Boeun **12** Mulan Valley, Sangcheon-myeon, Yeongdong **13** Minjoojisan Recreational Forest, Yonghwa-myeon, Yeongdong **14** Unjangsan Recreational Forest, Jeongcheon-myeon, Jinan **15** Pyunbaeg Recreational Forest, Samdong-myeon, Namhae.

Collections for this work were made during field trips of the last author in collaboration with colleagues from the National Institute of Forest Science (former Korea Forest Research Institute) in the margin of two larger inventory projects: “Diversity and molecular taxonomy of marasmielloid and gymnopoid fungi (Basidiomycota, Omphalotaceae) in South Korea” and “Phylogeny of litter decomposing fungi in South Korea”. The various localities in which *Cantharellus* specimens were collected are shown below (Fig. 1).

### Morphology

Macroscopic descriptions of collected specimens were based on fresh basidiomata. Colour abbreviations follow Kornerup and Wanscher (1983). Microscopic features were studied using dried material mounted in H_2_O, approximately 5% KOH, Melzer’s reagent and Congo Red, using an Olympus BX-50 light microscope (Tokyo, Japan) at 1000× magnification. For the hymenophore, “L” refers to the number of whole gill folds, while “l“ refers to the number of shorter gill folds between each pair of entire gill folds. For basidiospores, the factor E indicates the quotient of the length and width in any one basidiospore and Q is the mean of the E-values; the basidiospore values are based on 20 measurements in each collection. Specimens are preserved in the herbarium of the Moravian Museum, Brno, Czech Republic (**BRNM**) and duplicates deposited in the fungarium of the Natural History Museum in Paris, France (**PC**).

### Taxon sampling, sequence data and phylogenetic analyses

**Figure 2.**
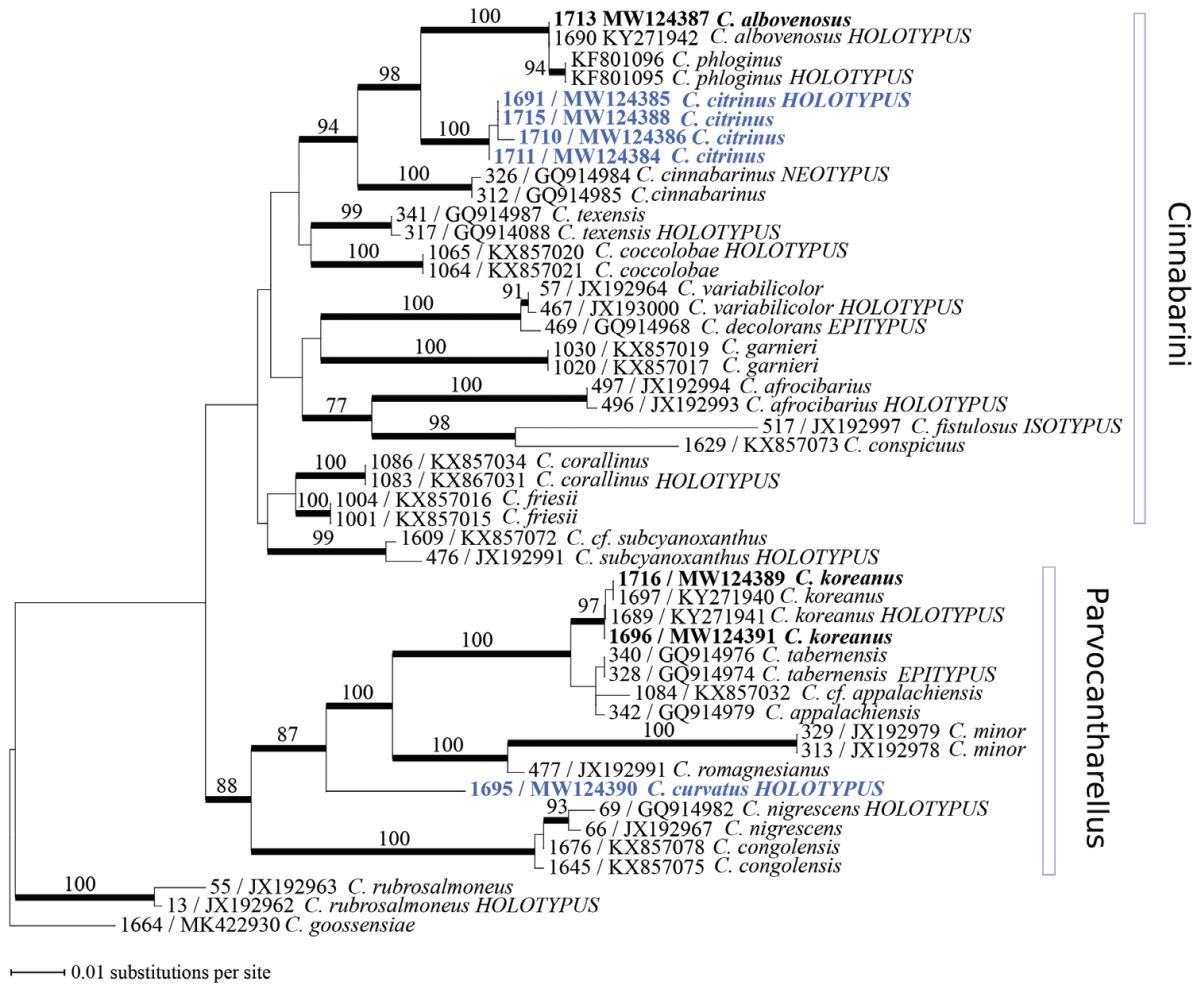
Most likely tree (-ln = 3254.82124) obtained by phylogenetic analyses of 48 *tef*-1 *Cantharellus* sequences. Supported branches are in bold with bootstrap values, when significant (≥ 70%), indicated along the branches. Sequences newly obtained for this study are in bold and highlighted in blue for the new species described in the present study. Extraction numbers and GenBank accession numbers for *tef*-1 sequences are reported before taxon names. Delimitation of *Cantharellus* subgenera *Cinnabarini* and *Parvocantharellus* (sensu lato) are indicated and *C. goossensiae* Heinem. and *C. rubrosalmoneus* (Buyck & V. Hofst.) Buyck & V. Hofst. (both in C. subg. Pseudocantharellus) are used as outgroup.

Translation elongation factor 1-alpha (*tef*-1) sequence data were produced following Buyck et al. (2014) for the newly described species from dried materials: four sequences for four collections of *Cantharellus citrinus* sp. nov and one sequence from a collection of *C. curvatus* sp. nov. Additional *tef*-1 sequences were obtained for two previously described species: for two collections of *C. koreanus* Buyck, Antonín & V. Hofst. and for one collection of *C. albovenosus* Buyck, Antonín & V. Hofst. We introduced these newly produced *tef*-1 sequences in the alignment obtained by Antonín et al. (2017). Species of subgenus Pseudocantharellus Eyssart. & Buyck were used as outgroup sequences. GenBank submissions numbers are given in Fig. 2. Alignment of sequence data was performed manually in MacClade (Maddison and Maddison 2003). Three independent searches for the most likely tree were conducted in PhyML v. 3.0 (Guindon and Gascuel 2003) to check for convergence toward the same likelihood value. These searches used the GTR evolutionary model (Abadi et al. 2019) with the proportion of invariable sites, the gamma shape parameter and the number of substitution categories estimated during the search. Branches were considered as significantly supported when maximum likelihood bootstrap support (ML-bs) was ≥ 70%.

## Results

### Phylogeny

The final alignment of *tef*-1sequences included 837 characters. After the removal of three spliceosomal introns, the alignment used for phylogenetic analyses included 629 characters. The most likely tree (Fig. 2) suggests that *C. citrinus* sp. nov. is part of subgenus Cinnabarini Buyck & V. Hofst. This species has a sister relationship (ML-bs = 98%) with the subclade (ML-bs =100%) including *C. albovenosus* and *C. phloginus* S.C. Shao & P.G. Liu. Our phylogeny further suggests that *C. curvatus* sp. nov. nests in the significantly supported subgenus Parvocantharellus Eyssart. & Buyck (ML-bs = 88%). The new species occupies a basal position in a subclade (ML-bs = 87%) composed of *C. romagnesianus* Eyssart. & Buyck, *C. minor* Peck, *C. appalachiensis* R.H. Petersen, *C. tabernensis* Feibelman & Cibula and *C. koreanus* and is clearly separated (ML-bs = 100%) from these other species. The only other subclade (ML-bs 100%) in the subgenus is composed of the blackening *Cantharellus* from tropical Africa, *C. nigrescens* Buyck & V. Hofst. and *C. congolensis* Beeli.

### Taxonomy

#### Cantharellus citrinus

Taxon classificationFungiScleractiniaFungiidae

Buyck, R. Ryoo & Antonín sp. nov.

42BEA16D-8A4A-53DC-9B24-5697BDC3ACE9

837726

Figs 3 , 4

##### Diagnosis.

Differs from its closest Asian and North American relatives in the variously coloured but often bright lemon yellow pileus, similarly tinted stipe and smaller size, as well as in differences in sequence data produced for the transcription elongation factor (*tef*-1).

**Figure 3.**
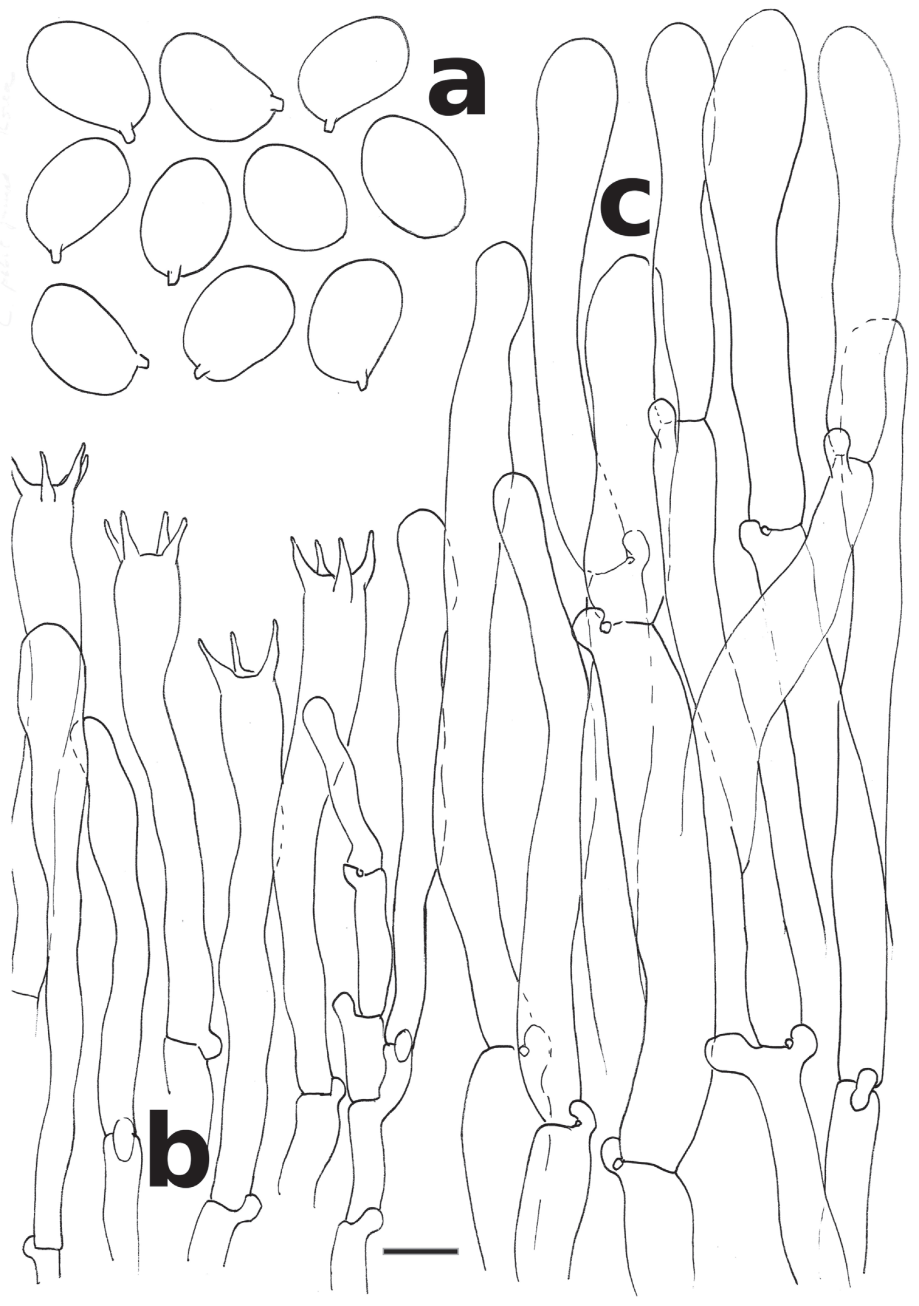
*Cantharellus citrinus* (holotype) **a** spores **b** basidia and basidiola **c** hyphal extremities of the pileipellis near mid-radius. Scale bar: 10 μm, but only 5 μm for spores. Drawings B. Buyck.

**Figure 4.**
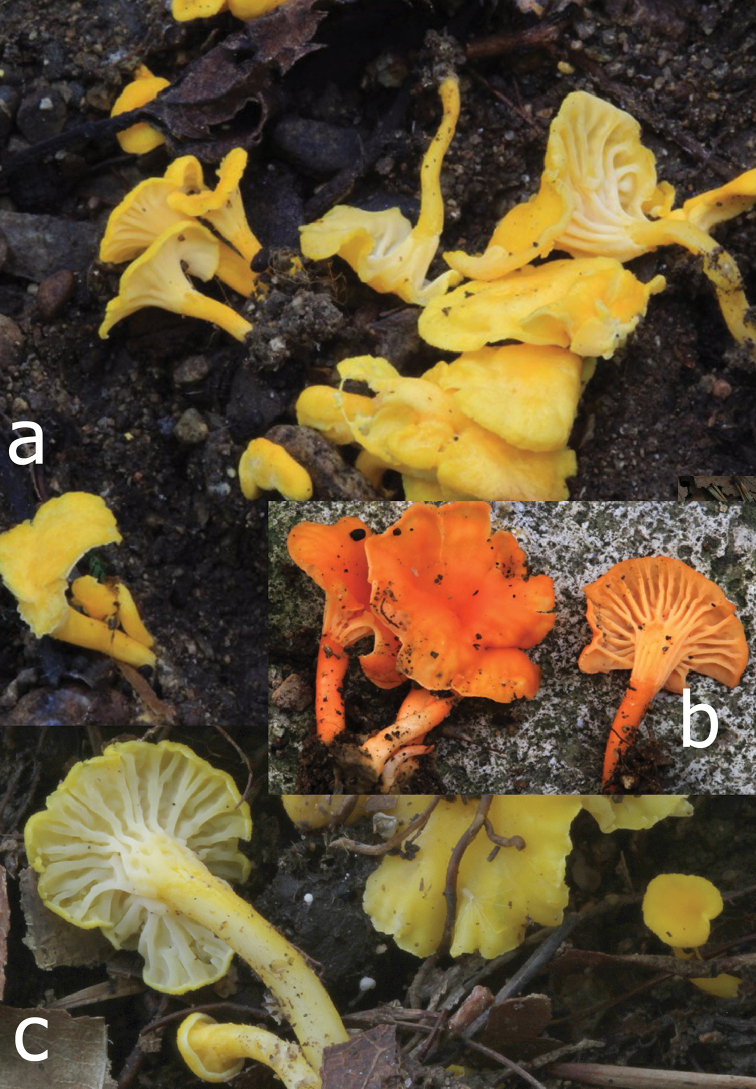
**a–c***Cantharellus citrinus*, yellow, more common form **a** (VA 13.170) **b***C. citrinus*, reddish orange form (VA 16.169) **c** (VA 13.156, holotype). Photos V. Antonín.

##### Holotype.

South Korea. Geoi-san, Cheong-cheon-myeon, alt. 330 m, 36°37'02.99"N, 127°49'36.56"E, 14 Aug 2013, V. Antonín, R. Ryoo & K.-H. Ka, 1691 / VA 13.156 (holotype: BRNM 825748; isotype: PC 0142457).

##### Description.

Basidiomata dispersed in small groups or fascicles. Pileus 4–20 mm broad, convex, with involute margin when young, then plane or infundibuliform with depressed centre and inflexed to straight, smooth margin, irregularly undulate when old, hygrophanous, finely tomentose when very young, soon glabrescent and smooth or slightly rugulose, uniformly coloured, light yellow, orange yellow to light orange (3–4A6, 4–5A5–7), sometimes with greyish yellow tinge when old. Hymenophore composed of thick vein-like folds, sometimes strongly decurrent in a reticulate pattern on upper stipe, often not reaching the pileus margin, forking or with rare lamellulae, transversely anastomosed in between, white to whitish (3A2); edges concolorous. Stipe 4–22 × 1–3(–4) mm, slightly tapering towards base when young, then cylindrical, sometimes curved, finely pubescent when young, later glabrescent, smooth, concolorous with pileus or slightly paler. Context thin, yellowish, fibrillose-hollow and yellowish whitish in stipe when old, with a spicy apricot smell and mild taste. Spore print not obtained.

Basidiospores ellipsoid, (7.3–)7.6–**8.24**–8.4(–8.8) × (5.1–)5.4–**5.67**–5.9(–6.1) μm, Q = (1.32–)1.34–**1.42**–1.50(–1.56), smooth, thin-walled. Basidia mostly (42–)66–80 × 8 μm, 4–5(–6)-spored, narrowly clavate; basidiola subcylindrical and slender when young, undulate-wavy in outline, later becoming narrowly clavate, subfusoid, sometimes irregular, rarely submoniliform, thin-walled. Cystidia not observed. Subhymenium composed of narrow, filamentous and cylindrical cells. Pileipellis a cutis composed of cylindrical, ± thin-walled, smooth or minutely incrusted, sparsely septate, (4–)8–12 μm wide hyphae; terminal cells (36–)50–110 × 4.0–15 μm, appressed to suberect, mostly slightly clavate, some with a subapical weak constriction, obtuse, thin-walled. Stipitipellis a cutis of cylindrical, slightly thick-walled, 2.5–6.0(–7.0) μm wide hyphae with isolated terminal cells distinct only in a narrow zone at very top, otherwise rare to absent, 20–51 × 4.0–11 μm, (narrowly) clavate, cylindrical or subfusoid, thin-walled. Clamp connections everywhere and distinct.

##### Habitat.

On soil near *Quercus mongolica* Fisch. ex Ledeb., *Q. acutissima* Carruth., *Quercus* sp., *Castanea crenata* Siebold & Zucc., *Carpinus laxiflora* (Siebold & Zucc.) Blume and *Abies koreana* E.H. Wilson.

##### Etymology.

The name refers to the frequent bright lemon yellow colour of pileus and stipe surface of the most common form.

##### Other specimens examined.

Jinan, Jeongcheon-myeon, Unjangsan Recreational Forest, alt. 390 m, 35°54'01.13"N, 127°24'59.41"E, 7 Sep 2016, V. Antonín, R. Ryoo, K.-H. Wang & Y.-S. Jang, 1710 / VA 16.169 (BRNM 825753, PC 0142467). Ibid., 1711 / VA 16.170 (BRNM 825754, PC 0142468). Yeongdong, Yonghwa-myeon, Minjoojisan Recreational Forest, alt. 540 m, 36°03'14.57"N, 127°49'43.15"E, 26 Aug 2015, V. Antonín, K.-H. Ka, K.S. Kim & J.A. Kang, 1715 / VA 15.93 (PC 0142472).

##### Remarks.

The description is based on the type specimen, but examination of the other specimens shows that variation of morphological features includes a rather wide amplitude of the overall colour, which seems – based on identical *tef1* sequences – to extend from entirely and predominantly pale lemon yellow to an overall deep orange. Collection from Jinan (VA 16.169, BRNM 825753, PC 0142467) differs from other collections of this species by an orange (5–6A7) pileus, light yellow to light orange (4–5A5) lamellae and a stipe more or less concolorous with the pileus.

This new species is here placed in Cantharellus subg. Cinnabarini (Fig. 2), a subgenus that comprises several species exhibiting a similarly wide colour range, e.g. the Malagasy *C. variabilicolor* Buyck & V. Hofst. (in Ariyawansa et al. 2015) or the North American *C. cinnabarinus* (Schwein.) Schwein. *Cantharellus citrinus* is here shown to be part of a well-supported clade composed of two other Asian species, the Chinese *C. phloginus* and Korean *C. albovenosus*. The latter two species are very different in general aspect, but, except for a single mutation in the coding part, the *tef*-1 sequences of both species are identical, even including the introns. Yet, their very different general habitus justifies us in our view that we should accept them as a separate species. The clade comprising these Asian species is sister to a clade composed of North American species.

Because of its very small overall size and comparable overall colour, *C. citrinus* could also easily be mistaken for some species in Cantharellus subg. Parvocantharellus Eyssart. & Buyck, in particular the European *C. romagnesianus* (= *C. pseudominimus* Eyssart. & Buyck, see Olariaga et al. 2015). Under the microscope, *C. citrinus* differs hardly from its Asian relatives and identification relies principally on field characters or sequence data.

#### Cantharellus curvatus

Taxon classificationFungiScleractiniaFungiidae

Buyck, R. Ryoo & Antonín sp. nov.

D4E48F69-48DF-582A-A2A0-77497D352A91

837727

Figs 5 , 6

##### Diagnosis.

Differs from the European *C. romagnesianus* in the distinctly smaller spores and shorter basidia (see Olariaga et al. 2016), the strongly veined hymenophore and sequence data obtained from the transcription elongation factor one alpha (*tef-1*).

**Figure 5.**
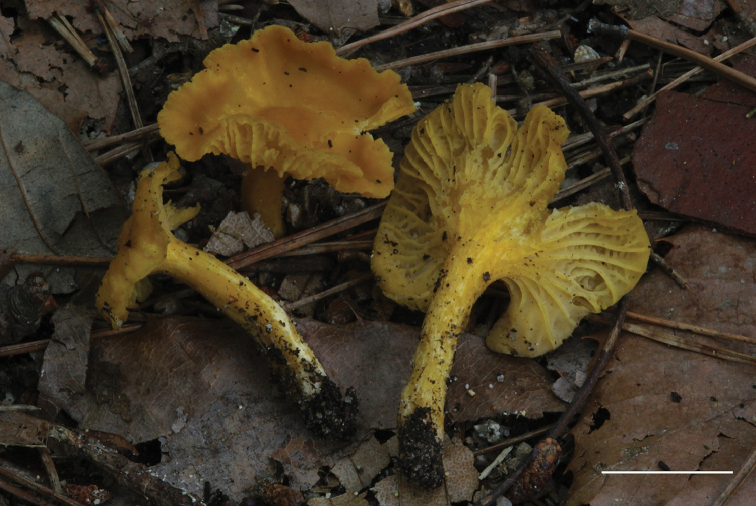
*Cantharellus curvatus* (VA 14.57, holotype). Scale bar: 15 mm. Photos V. Antonín.

##### Holotype.

South Korea. Yesan, Deoksan-myeon, Sudeok-sa, alt. 220 m, 36°39'57.40"N, 126°37'20.91"E, 8 Jul 2014, V. Antonín & K.-H. Ka, 1695 / VA 14.57 (holotype: BRNM 825749; isotype: PC0142461)

##### Description.

Basidiomata in groups. Pileus 20–30 mm broad, low convex with a low broad central umbo and involute margin, then irregularly applanate or shallowly infundibuliform with an undulate, often uplifted margin, hygrophanous, not translucently striate, smooth, glabrous, watery dull yellow when moist, drying out to orangish yellow (± slightly more yellow than 4A5). Hymenophore composed of distant gill folds [L = 37–40], shortly decurrent, thick, sometimes furcate when young, furcate-anastomosed in upper half when old, pale yellow (± 3A3), ± dirty (greyish) yellow at the end; edge concolorous. Stipe 25–30 × 3.5–4 mm, cylindrical and tapering towards base, longitudinally fibrillose, yellow (± concolorous with pileus). Context pale whitish-yellowish, with cantharelloid smell.

Basidiospores (7.25–)7.5–**8.05**–9.0 × 5.0–**5.25**–6.0(–6.25) μm, Q =1.40–**1.52**–1.66, ellipsoid, rarely broadly ellipsoid, ventrical applanate or suballantoid, thin-walled, smooth. Basidia 42–55 × 9.5–12 μm, (4–)6-spored, narrowly clavate, clamped. Basidiola 15–42 × 3.0–11 μm, clavate, cylindrical, subfusoid, irregularly curved or undulate. Trama hyphae of cylindrical to fusoid, clamped, ± thin-walled, 4.0–20 μm wide cells. Pileipellis a cutis composed of cylindrical, clamped, mostly thin-walled, 4.0–10 μm wide hyphae; terminal cells appressed to suberect, mostly cylindrical, slightly thick-walled, up to 80 μm long and 5.0–10(–15) μm wide. Stipitipellis a cutis of cylindrical, parallel, slightly thick-walled, clamped, 3.0–6.0 μm wide hyphae. Terminal cells appressed to suberect, clavate or cylindrical.

##### Habitat.

On soil under *Pinus densiflora* Siebold & Zucc. and *Castanea crenata*.

##### Etymology.

Referring to the curved-undulate hymenial cells, viz. basidia and particularly basidiola.

##### Remarks.

This Asian species differs from the European *C. romagnesianus*, presently the most similar chanterelle, in the distinctly smaller spores and shorter basidia (see Olariaga et al. 2016), further also in the strongly anastomosing hymenophore and in sequence data obtained from the transcription elongation factor one alpha (*tef-1*).

**Figure 6.**
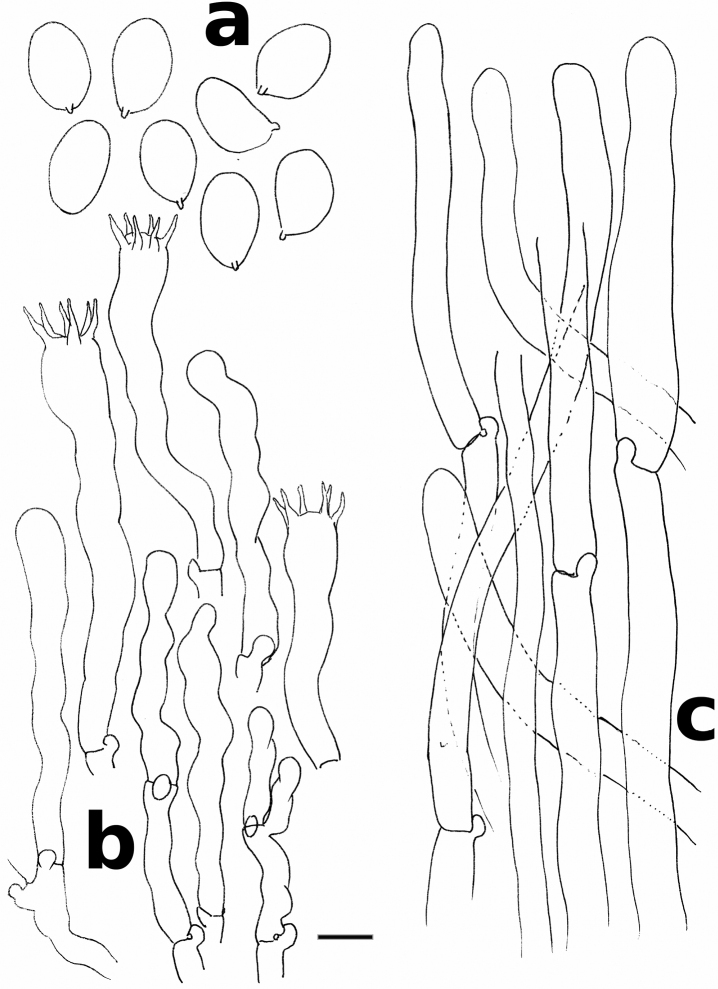
*Cantharellus curvatus* (holotype) **a** spores **b** basidia and basidiola **c** hyphal extremities of the pileipellis near mid-radius. Scale bar: 10 μm, but only 5 μm for spores. Drawings B. Buyck.

#### Cantharellus anzutake

Taxon classificationFungiScleractiniaFungiidae

W. Ogawa, N. Endo, M. Fukada & A. Yamada, Mycoscience 59(2): 158 (2018)

AB6ECAFE-E792-561F-A8CC-A450442149B1

Figs 7 , 8 , 9

##### Description.

Pileus 10–40 mm broad, convex-conical when young, soon plane to broadly funnel-shaped, sometimes with a low obtuse umbo at centre, margin involute then inflexed to straight and undulate, pruinose when young then ± glabrous, greasy when moist, smooth or slightly uneven, not translucently striate, yellow (4A7–8), sometimes with darker (“dirty”) centre. Lamellae moderately close, L = c. 25–30, decurrent, often furcate, rarely branched, whitish to pale cream from ± half radius toward the stipe attachment, then yellow towards pileus margin. Stipe 20–40 × 3.5–6 mm, cylindrical, not broadened towards base, finely pruinose when young, then glabrous, white, not hollowing. Context yellow beneath pileipellis, white otherwise. Smell slight, cantharelloid. Taste mild with slightly sharp aftertaste. Spore print not obtained.

**Figure 7.**
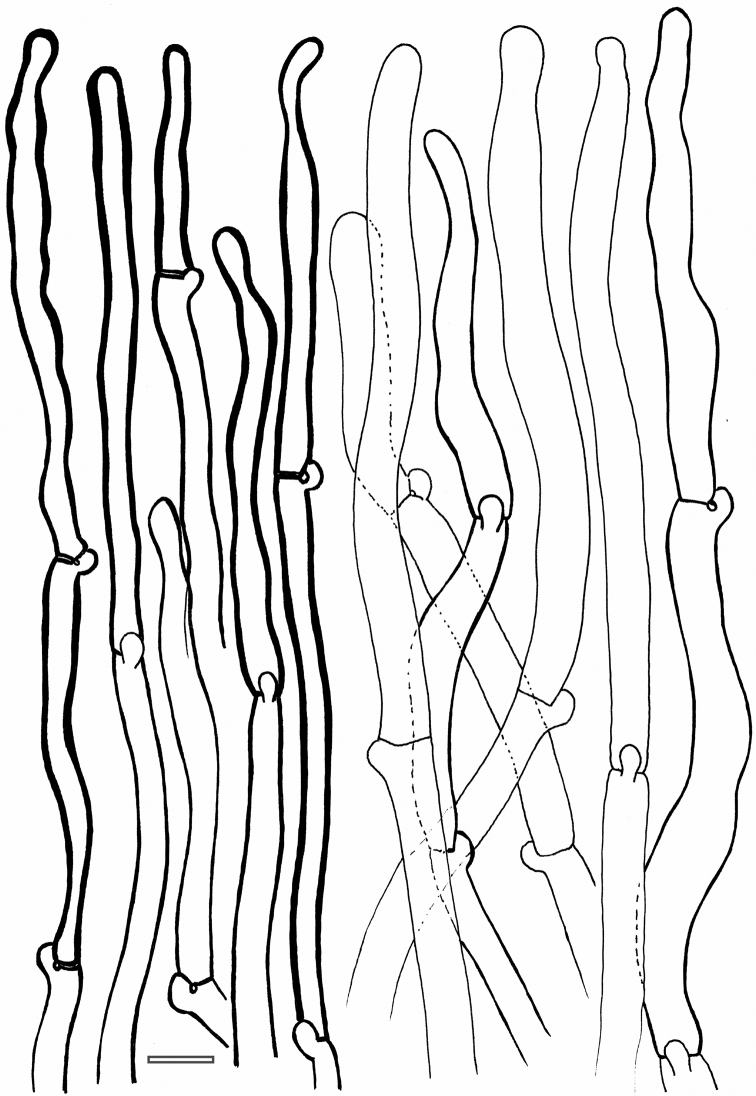
*Cantharellus anzutake*, microscopic features. Hyphal extremities at the pileus surface, on the left near the pileus center, on the right closer to the pileus margin. Scale bar: 10 μm. Drawings B. Buyck.

Basidiospores ellipsoid to ovoid, (6.9–)7.2–**7.56**–8.0(–8.3) × (4.6–)4.8–**5.10**–5.4(–5.6) µm, Q = (1.31–)1.39–**1.49**–1.58(–1.68), smooth, with a small apiculus. Basidia clavate-pedicellate, (60–)70–80 × 7–8 µm, long and slender, mostly 6(–5)-spored with stout sterigmata. Subhymenium filamentous, composed of long and slender, cylindrical cells of similar diam. as the basidium base. Cystidia none. Pileipellis a loose tissue of intricately intertwining, sparsely septate, long and slender hyphal ends, near the pileus margin often aggregated in long tufts; hyphal ends composed of long, cylindrical, 5–8(–12) µm diam. cells, with refringent, thin- to slightly thickened walls, but in the pileus centre more frequently thick-walled; the terminal cell slender, toward the pileus margin (40–)60–130 µm long, obtuse rounded at the tip, cylindrical, hardly differentiated from subapical ones; in the pileus centre often somewhat irregularly constricted near the tip, but never very strongly so, and usually shorter, 30–100 µm, and on average somewhat narrower.

**Figure 8.**
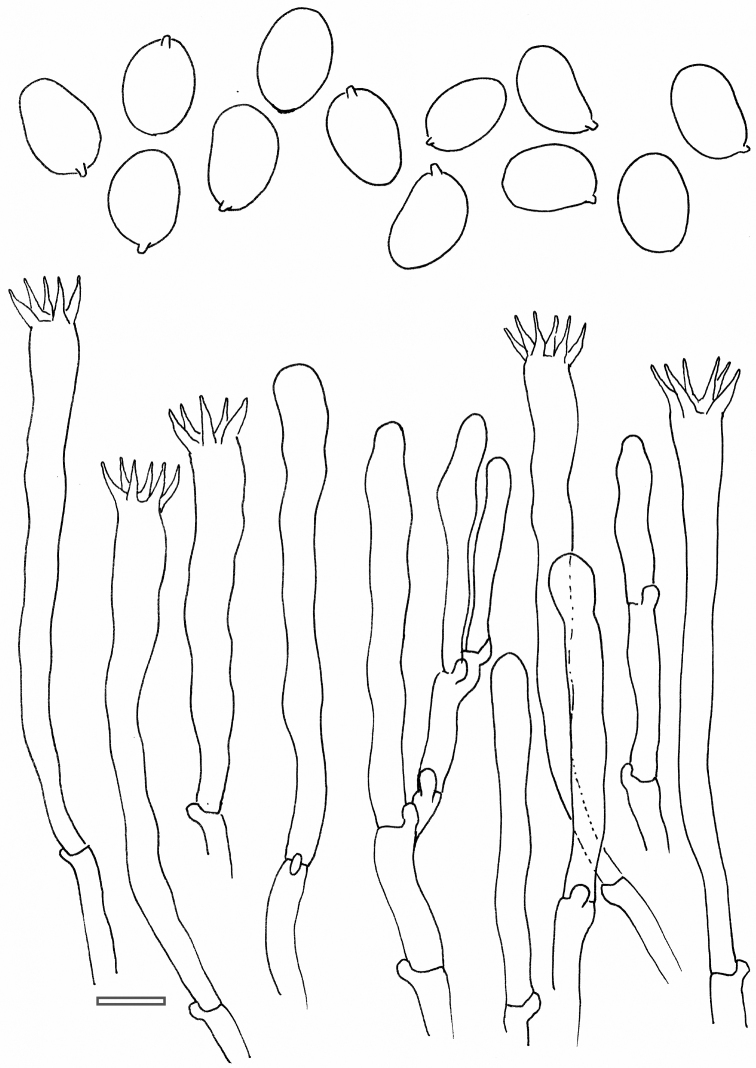
*Cantharellus anzutake*. Microscopic features. Basidia, basidiola and spores. Scale bar: 10 μm, but only 5 μm for spores. Drawings B. Buyck.

##### Habitat.

On soil under *Pinus densiflora*, *Carpinus laxiflora* and *Quercus mongolica*.

##### Specimens examined.

Jinan, Jeongcheon-myeon, Unjangsan Recreational Forest, 35°54'05.55"N, 127°24'53.89"E, alt. 400 m, 31 Aug 2016, V. Antonín, K.-H. Ka & S.-H. Kim, 1708 / VA 16.140 (BRNM 825751, PC 0142465). Ibid., VA 16.142 (BRNM 825752, PC 0142466).

**Figure 9.**
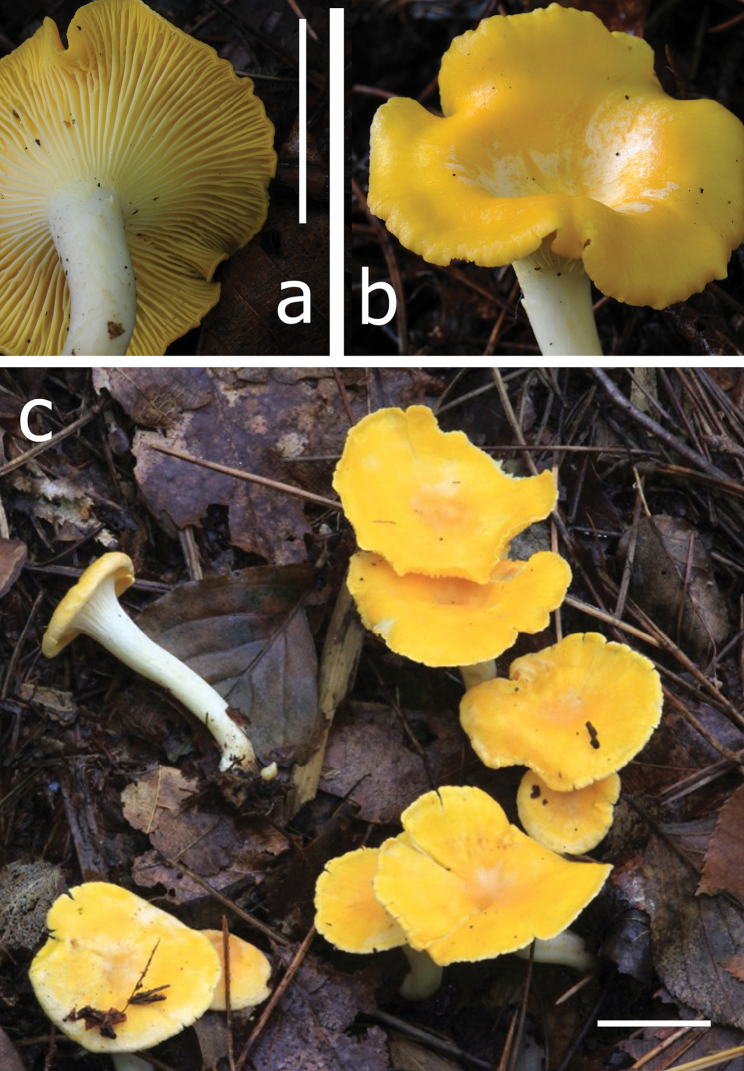
*Cantharellus anzutake***a, b** Ka & Ryoo 3_Korea_1-2 [22/08/2012, Pyeongchang, Jungwangsan, 37°27'27.48"N, 128°29'04.35"E, 771 m asl, under *Pinus koraiensis* Siebold & Zucc.] **c** Antonín 16.140 (PC0142465). Note the very pale hymenophore in young specimens, remaining for a long time paler closer to the stipe when maturing. Scale bar: 20 mm.

##### Remarks.

This species is a typical member of Cantharellus subg. Cantharellus, and belongs to a group that is often referred to as the ‘golden chanterelles’ or the *C. cibarius* Fr. complex, representing the commercially most important chanterelles on the international market. This species complex is reputedly very difficult to identify, in particular because of the very variable field aspect of the various species involved (Olariaga et al. 2015, 2016). Hence, positive identification frequently requires molecular sequence data. Our identification is here based on the high quality ITS sequence we obtained for VA 16.142 and which is identical to the one deposited for the *C. anzutake* holotype (GenBank LC085359, similarity 100% for 100% coverage); both these ITS differ from other yellow species of chanterelles described from Asia by a ca 100 bp deletion in the ITS1 region (Ogawa et al. 2018).

Because of the whitish hymenophore when young and the sometimes deep orange-yellow to cinnamon buff pileus surface, this species may be somewhat reminiscent of *C. albovenosus*. The latter species, however, has always a much brighter orange pileus and a more veined hymenophore that remains white, even with age, and it belongs in subgenus Cinnabarini (see Antonín et al. 2017). It is interesting to note that both Japanese and Korean specimens were collected near *Pinus densiflora* among possible host trees.

## Supplementary Material

XML Treatment for Cantharellus citrinus

XML Treatment for Cantharellus curvatus

XML Treatment for Cantharellus anzutake
